# Mapping From Visual Acuity to EQ-5D, EQ-5D With Vision Bolt-On, and VFQ-UI in Patients With Macular Edema in the LEAVO Trial

**DOI:** 10.1016/j.jval.2020.03.008

**Published:** 2020-07

**Authors:** Becky M. Pennington, Mónica Hernández-Alava, Philip Hykin, Sobha Sivaprasad, Laura Flight, Abualbishr Alshreef, John Brazier

**Affiliations:** 1 School of Health and Related Research, University of Sheffield, Sheffield, England, UK; 2 National Institute for Health Research Moorfields Biomedical Research Centre, London, England, UK

**Keywords:** bolt-off, bolt-on, crosswalk, EQ-5D, EQ-5D vision, mapping, visual acuity, VFQ, VFQ-UI

## Abstract

**Objectives:**

Mappings to convert clinical measures to preference-based measures of health such as the EQ-5D-3L are sometimes required in cost-utility analyses. We developed mappings to convert best-corrected visual acuity (BCVA) to the EQ-5D-3L, the EQ-5D-3L with a vision bolt-on (EQ-5D V), and the Visual Functioning Questionnaire-Utility Index (VFQ-UI) in patients with macular edema caused by central retinal vein occlusion.

**Methods:**

We used data from Lucentis, Eylea, Avastin in vein occlusion (LEAVO), which is a phase-3 randomized controlled trial comparing ranibizumab, aflibercept, and bevacizumab in 463 patients with observations at 6 time points. We estimated adjusted limited dependent variable mixture models consisting of 1 to 4 distributions (components) using BCVA in each eye, age, and sex to predict utility within the components and BCVA as a determinant of component membership. We compared model fit using mean error, mean absolute error, root mean square error, Akaike information criteria, Bayesian information criteria, and visual inspection of mean predicted and observed utilities and cumulative distribution functions.

**Results:**

Mean utility scores were 0.82 for the EQ-5D-3L, 0.79 for the EQ-5D V, and 0.88 for the VFQ-UI. The best-fitting models for the EQ-5D and EQ-5D V had 2 components (with means of approximately 0.44 and 0.85), and the best-fitting model for VFQ-UI had 3 components (with means of approximately 0.95, 0.74, and 0.90).

**Conclusions:**

Models with multiple components better predict utility than those with single components. This article provides a valuable addition to the literature, in which previous mappings in visual acuity have been limited to linear regressions, resulting in unfounded assumptions about the distribution of the dependent variable.

## Highlights

• Adjusted, limited dependent variable mixture models with multiple components better predict utility using the EQ-5D-3L with and without vision bolt-on and Visual Functioning Questionnaire-Utility Index (VFQ-UI) than mappings with only 1 component. Previous mappings in visual acuity have considered only linear regression using ordinary least squares (1 component).
• Our mappings can be used to predict 3 different measures of utility as a function of age, sex, and best-corrected visual acuity in both eyes. These variables are routinely included in economic evaluations of visual disorders.

## Introduction

Mappings to convert clinical measures to health utilities may be required for economic evaluations in which either health utilities were not reported in the clinical effectiveness studies or in which there is a need to relate modeled clinical outcomes to health utilities in the long-term. Health utilities may be generated using generic preference-based measures such as the EQ-5D or more specific measures such as the Visual Functioning Questionnaire-Utility Index (VFQ-UI) in visual disorders.

A multicenter phase 3 double-masked randomized controlled noninferiority trial comparing intravitreal therapy with ranibizumab (Lucentis) versus aflibercept (Eylea) versus bevacizumab (Avastin) for macular edema due to central retinal vein occlusion (CRVO; LEAVO) was a three-arm study of 463 patients over a 100-week period.^1^ The cost-effectiveness analysis comprised 2 parts: an economic evaluation alongside the clinical trial and an economic model to analyze the cost-effectiveness over a longer time horizon. LEAVO included 3 preference-based measures of utility: the EQ-5D five level, the EQ-5D five level with vision bolt-on (EQ-5D V), and the VFQ-UI. The EQ-5D five level with and without the vision bolt-on were converted to the 3-level version using the crosswalk of van Hout et al^2^ as recommended by the National Institute for Health and Care Excellence (NICE) in its methods guide.^3^ In the economic analysis, these measures could be used directly in the within-trial analysis. The economic model predicted visual acuity until all patients had died and thus required a mapping to relate visual acuity to health utility.

The EQ-5D asks patients to rate their health across 5 dimensions: mobility, self-care, usual activities, pain/discomfort, and anxiety/depression. In the 3-level (3L) version, each dimension has 3 levels: no problems, some problems, and extreme problems.^4^ In the 5-level (5L) version, each dimension has 5 levels: no problems, slight problems, moderate problems, severe problems, and extreme problems.^5^ The patient’s responses to the 5 dimensions are combined into a single-digit number representing the patient’s health state. A sample of 3L health states was valued using a representative sample of the UK population using the time trade-off method, and regression models were used to generate a tariff of values for all health states.^6^ A value set has also been produced for the EQ-5D-5L,^7^ although quality assurance has raised concerns about the data.^8^

A bolt-on exists for the EQ-5D, which also asks patients about their vision. In the 5L version, there are 5 levels: no problems, slight problems, moderate problems, severe problems, and extreme problems. In the 3L version, there are 3 levels: no problems, some problems, and extreme problems. A scoring algorithm exists for the vision bolt-on to the EQ-5D-3L, whereby a decrement of 0.0378 is subtracted from the EQ-5D-3L score for patients who report some problems with vision and a decrement of 0.130 is subtracted from the EQ-5D-3L score for patients who report extreme problems with vision.^9^

The VFQ-UI was developed from the National Eye Institute Visual-Function Questionnaire-25, which contains 25 items with 5 or 6 response levels. The VFQ-UI asks patients to rate their health across 6 dimensions. Near vision, social vision, and distance vision each have 4 levels relating to the level of difficulty (no, little, moderate, or extreme) that the patient has in specific activities. Role difficulty asks whether the patient is not limited, limited a little of the time, limited some of the time, or limited in how long they can work or do other activities. Vision dependency asks whether the patient does not have to stay home or has to stay home some, most, or all of the time. Mental health asks whether the patient does not worry or worries some, most, or all of the time about doing this, which will embarrass themselves or others.^10^ A sample of VFQ-UI health states was valued using participants from Australia, Canada, the United Kingdom, and the United States using the time trade-off method, and regression models were used to develop a scoring algorithm for all potential health states.^11^

The primary outcome in LEAVO was the change in best-corrected visual acuity (BCVA) of the study eye measured using early treatment for diabetic retinopathy study (ETDRS) letter score from baseline to 100 weeks. The ETDRS chart consists of 14 rows of 5 letters, with an equal difficulty on each row but with decreasing size. For patients who could read 20 or more letters correctly at 4m, their BCVA score was the number of letters read at 4m plus 30. For patients who could read fewer than 20 letters correctly at 4m, their BCVA score was calculated as the total number of letters read correctly at 4m plus the total number of letters read correctly at 1m in the first 6 lines.^12^ A higher BCVA score indicates better vision. The economic model used BCVA letter scores in both the study and nonstudy eye to model disease progression. To model quality of life, BCVA ETDRS letter scores were therefore linked to EQ-5D, EQ-5D V, and VFQ-UI. Previous research has shown that BCVA letter scores in both eyes are important in predicting utility.^13^, ^14^, ^15^, ^16^ We aimed to develop a mapping to predict EQ-5D, EQ-5D V, and VFQ-UI scores from the BCVA score of the study and nonstudy eye.

## Methods

### Data

LEAVO collected EQ-5D-5L, vision bolt-on, and VFQ-UI responses in addition to BCVA ETDRS scores for both eyes (among other measures) at weeks 0, 12, 24, 52, 76, and 100. We used the van Hout crosswalk^2^ (consistent with guidance from NICE^17^) to convert the EQ-5D-5L responses to EQ-5D-3L scores. We valued the EQ-5D V scores by subtracting 0.0378 for patients with level or moderate vision problems and 0.130 for patients with severe or extreme vision problems.^9^ We used the VFQ-UI scoring system described by Rentz et al.^11^ We combined data from all 6 visits to maximize the number of observations. At each observation, we generated variables for the better-seeing eye (BSE) and worse-seeing eye (WSE) according to whether the BCVA score was higher in the study or nonstudy eye.

### Statistical Analysis

The EQ-5D-3L has a distinctive distribution, with a lower bound of –0.594 and an upper bound of 1. It displays a multimodal distribution with a mass of observations at 1 and a gap between 1 and the next-best health state (0.906 when crosswalking 5L to 3L). These properties mean that standard statistical models are often a poor fit, and so we used bespoke models developed for modeling EQ-5D-3L: adjusted limited dependent variable mixture models (ALDVMMs).^18^ The ALDVMM is based on mixtures of bespoke distributions that accommodate the limits to the EQ-5D-3L distribution at full health, at the worst health state, and the gap between full health and the next feasible health state. In addition, being a mixture model, it provides a flexible semiparametric framework for modeling distributions with unusual shapes. Although initially developed for modeling the EQ-5D-3L, the model has been shown to be applicable to other preference-based measures.^19^ Because the EQ-5D V is calculated from the EQ-5D, it has a similar distribution. The VFQ-UI is scored between 0.343 and 0.956, and its distribution has different characteristics from that of the EQ-5D.

ALDVMMs are flexible models that feature multiple components; each component’s distribution has different parameters. Additional variables predict the probability of each observation belonging to each component. We estimated ALDVMMs with 1 to 4 components. We included BSE BCVA, WSE BCVA, and the interaction between them (to allow for the effect of BSE to vary with WSE); age; and sex as independent variables to predict the EQ-5D, EQ-5D V, or VFQ-UI within the components. We considered BSE and WSE BCVA as determinants of component membership. We did not include the trial arm as an independent variable because there was no statistically significant difference between utility in the 3 arms in the trial,^20^ and it is anticipated that an intervention would affect utility by affecting the BCVA score.

To compare results across models, we considered standard model fit measures/criteria such as mean error mean absolute error (MAE), root mean square error (RMSE), Akaike information criteria (AIC), Bayesian information criteria (BIC), and graphical methods for model selection in mapping.^19^ A mean error closer to zero and smaller MAE, RMSE, AIC, and BIC indicated a better fit. Nevertheless, standard measures based on “errors” (difference between the data and the model prediction) often provide conflicting results because they are based on different scoring functions. For example, RMSE penalizes the existence of large outliers more than MAE does. Both AIC and BIC are likelihood-based criteria with a penalty for model complexity, but the penalty BIC imposes tends to be larger, often resulting in AIC and BIC selecting models with different number of parameters. Because of these issues, graphical methods have been shown to be essential for mapping model selection. Specifically, we plotted the mean of the predicted utility scores with the mean observed values by BSE and WSE BCVA scores. We also simulated data from the models and plotted the cumulative distribution functions comparing simulated with observed data across the severity range. We followed good practice guidance produced by the International Society for Pharmacoeconomics and Outcomes Research.^21^

## Results

**Figure 1:**
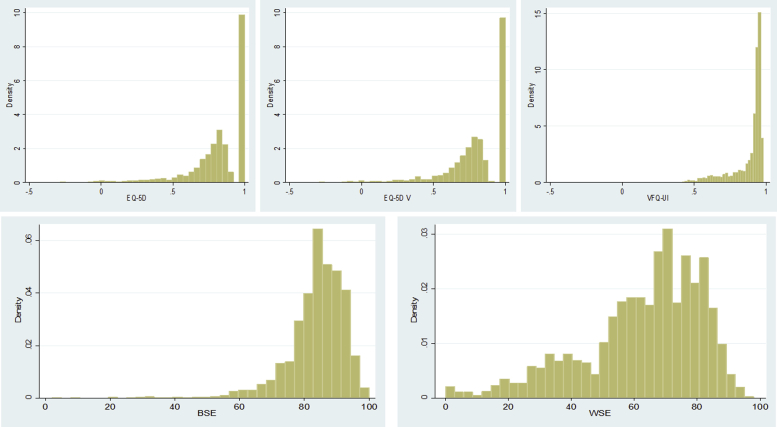
Distribution of the EQ-5D, EQ-5D V, and VFQ-UI baseline scores and the BSE and WSE baseline scores.

There were 2778 observations in total (6 time points for 463 patients). There were 2558 observations, which included data for age, sex, and BCVA in both eyes. Utility data were available for 2470 of these observations for the EQ-5D, 2321 for the EQ-5D V, and 2481 for the VFQ-UI. The distributions of EQ-5D, EQ-5D V, and VFQ-UI scores and for the BSE and WSE BCVA ETDRS scores are shown in Figure 1. The EQ-5D displayed its typical multimodal distribution, with a spike of 38.47% of observations at 1, a gap between 1 and 0.906, and peak around 0.8 and a long tail to the left. The EQ-5D V had a similar shape, with a spike at 1 (representing 18.55% of patient observations in full health according to the EQ-5D who have no vision problems) and a gap between 1 and the next-best health state (here, this is 0.962, as 19.46% of patients have full health according to the EQ-5D but mild vision problems). The values in the peak at about 0.8 and the tail were lower for the EQ-5D V than for the EQ-5D because of the decrements for vision problems. Because of vision decrements, there were slightly more observations less than 0 for the EQ-5D V than for the EQ-5D (1.38% vs 0.86%). The VFQ-UI displayed a highly skewed distribution, with a peak at about 0.9 and a long left tail, similar to its distribution in previous studies.^14^

BSE indicates better-seeing eye visual acuity; EQ-5D V, EQ-5D with vision bolt-on; VFQ-UI, Visual Functioning Questionnaire-Utility Index; WSE, worse-seeing eye visual acuity.

Descriptive statistics are shown in Table 1. Means and proportions for BCVA, age, and sex were calculated from the 2558 observations with these data available. Means for the utility measures excluded patients where utility data were not available. The Spearman correlation coefficients between the 3 utility measures and BSE, WSE, age, and sex (Appendix Table 1 in Supplemental Materials found at https://doi.org/10.1016/j.jval.2020.03.008) demonstrated a statistically significant correlation (at the 5% level) between the variables.

**Table 1:** Descriptive statistics of best corrected visual acuity, EQ-5D with and without the vision bolt-on and the Visual Functioning Questionnaire-Utility Index from the LEAVO study data.

Variable	Mean ± SD	Minimum	Maximum
EQ-5D index score	0.820 ± 0.211	−0.287	1
EQ-5D vision bolt-on score	0.792 ± 0.220	−0.295	1
VFQ-UI score	0.881 ± 0.115	0.400	0.980
Better-seeing eye BCVA score	83.795 ± 9.278	2	100
Worse-seeing eye BCVA score	62.664 ± 18.965	0	98
Age	70.279 ± 12.785	21.664	98.226
Male, n (%)	1,466 (57.31%)		

BCVA indicates best-corrected visual acuity; SD, standard deviation; VFQ-UI, Visual Functioning Questionnaire-Utility Index.

### Model Fit Statistics

#### EQ-5D

Table 2 presents the fit statistics for the ALDVMMs for EQ-5D. Within the 1- and 2-component models, adding an interaction term worsened the model fit, as demonstrated by increased AIC, BIC, MAE, and RMSE for the 2-component model. We therefore omitted this variable from models with further components. Within the 2-component model, using the BSE to predict component membership improved the model fit and additionally using the WSE further improved the model fit. We therefore chose between 1-, 2-, 3-, and 4-component models without the BSE∗WSE interaction term and using BSE and WSE to determine component membership. The 2-, 3-, and 4-component models had the same RMSE and similar MAE (slightly higher for the 3 and 4 components than 2 components), but the BIC was lowest for the 2-component model (model 6).

**Table 2:** Model fit statistics for the ALDVMMs for EQ-5D.

Model	Number of components	Within-component variables	Between-component variables	Log likelihood	AIC	BIC	Mean error	MAE	RMSE
1	1	BSE, WSE, age, sex	NA	−704.413	1420.8250	1455.6970	0.0039	0.1445	0.1987
2	1	BSE, WSE, BSE∗WSE, age, sex	NA	−704.412	1422.8240	1463.5080	0.0039	0.1445	0.1987
3	2	BSE, WSE, age, sex	Constant	−479.682	985.3648	1060.9200	−0.0017	0.1435	0.1986
4	2	BSE, WSE, BSE∗WSE, age, sex	Constant	−477.872	985.7431	1072.9230	−0.0013	0.1437	0.1988
5	2	BSE, WSE, age, sex	BSE	−476.116	980.2326	1061.6000	−0.0006	0.1436	0.1987
6	2	BSE, WSE, age, sex	BSE, WSE	−467.949	965.8980	1053.0780	−0.0007	0.1435	0.1982
7	3	BSE, WSE, age, sex	Constant	−456.618	953.2355	1069.4750	−0.0009	0.1438	0.1986
8	3	BSE, WSE, age, sex	BSE, WSE	−449.198	946.3959	1085.8830	−0.0009	0.1437	0.1982
9	4	BSE, WSE, age, sex	Constant	−441.025	936.0499	1092.9730	−0.0012	0.1437	0.1985
10	4	BSE, WSE, age, sex	BSE, WSE	−419.682	905.3647	1097.1600	−0.0003	0.1421	0.1960

AIC indicates Akaike information criteria; BIC, Bayesian information criteria; BSE, better-seeing eye visual acuity; MAE, mean absolute error; NA, not applicable; RMSE, root mean square error; WSE, worse-seeing eye visual acuity.

#### EQ-5D V

Supplementary Table 2 (in Supplemental Materials found at https://doi.org/10.1016/j.jval.2020.03.008) presents the fit statistics for the ALDVMM models for the EQ-5D V. As with the EQ-5D, the interaction term worsened the model fit, and using the BSE and WSE to predict component membership improved the model fit. The BIC was lowest for the 2-component model with component membership predicted by BSE and WSE, whereas the mean error, MAE, and RMSE were similar for the 2-, 3-, and 4-component models.

#### VFQ-UI

Supplementary Table 3 presents the fit statistics for the ALDVMM models for the VFQ-UI. We found that the 3-component model with component membership predicted by the BSE and WSE had the lowest BIC, whereas the mean error, MAE, and RMSE were similar between the 2-, 3-, and 4-component models.

### Comparison of Mean Predicted and Observed Utility Scores

**Figure 2:**
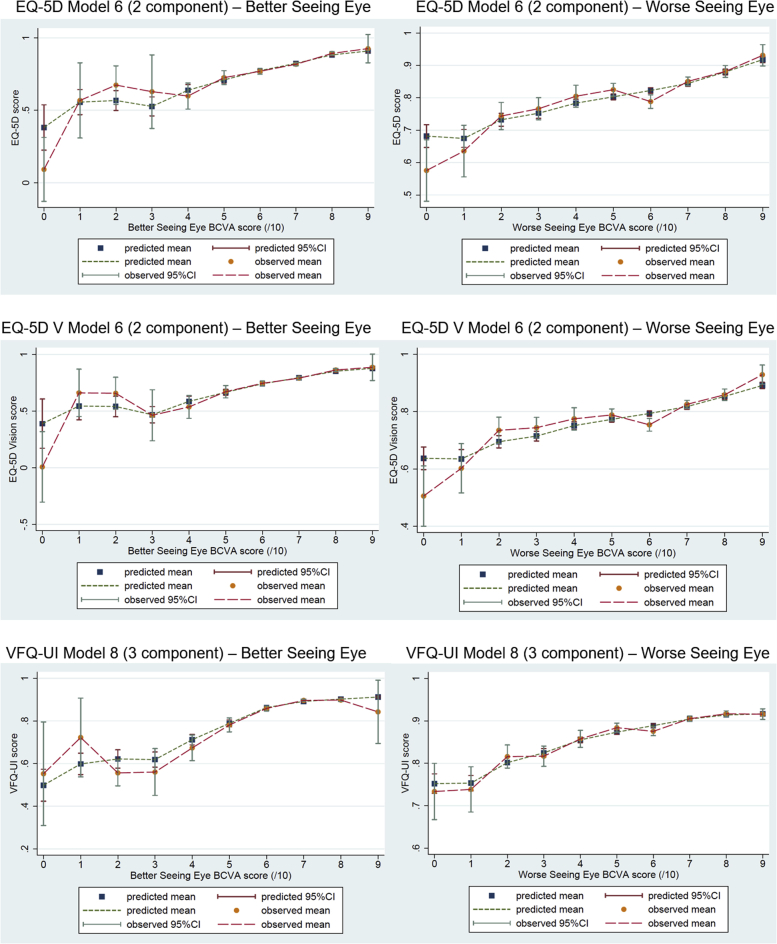
Mean predicted and observed utility scores for 2-component models for the EQ-5D and EQ-5D V and 3-component model for the VFQ-UI.

Figure 2 presents the mean predicted and observed utility scores for the 2-component models for the EQ-5D and EQ-5D V and the 3-component model for the VFQ-UI (all with within-component variables for BSE, WSE, age, and sex and with component membership predicted by BSE and WSE), against BSE and WSE scores. The graphs of the mean predicted and observed utility scores for models with a higher number of components (not presented here) did not indicate a better prediction and so did not change the decisions regarding the best-fitting models. Generally, we see that utility increases as BSE and WSE increase, but the observed values are nonmonotonic for BCVA scores less than 40. The observed utility values for BCVA scores less than 40 are nonmonotonic because of the small number of observations and the potential for visual acuity loss to be caused by other conditions such as retinal detachment and endophthalmitis in addition to CRVO. For all utility measures, the predicted values lie furthest from the observed values for very low BSE and WSE scores. For lower BSE and WSE scores, the confidence intervals for the utility scores are much wider than for higher BSE and WSE scores; this is because there are few observations at low BSE and WSE scores (see Figure 1). At higher BSE and WSE scores, the predicted mean values appear to lie very close to the observed mean values for all 3 utility measures. The mean predicted utilities lie closer to the mean observed scores for the VFQ-UI than for the EQ-5D and EQ-5D V (particularly at lower BSE and WSE scores). This is unsurprising when considering that the VFQ is focused on vision, whereas the EQ-5D and EQ-5D V also measure other elements of health-related quality of life.

BCVA indicates best-corrected visual acuity; CI, confidence interval; EQ5D V, EQ-5D with vision bolt-on; VFQ-UI, Visual Functioning Questionnaire-Utility Index.

### Cumulative Distribution Functions

**Figure 3:**
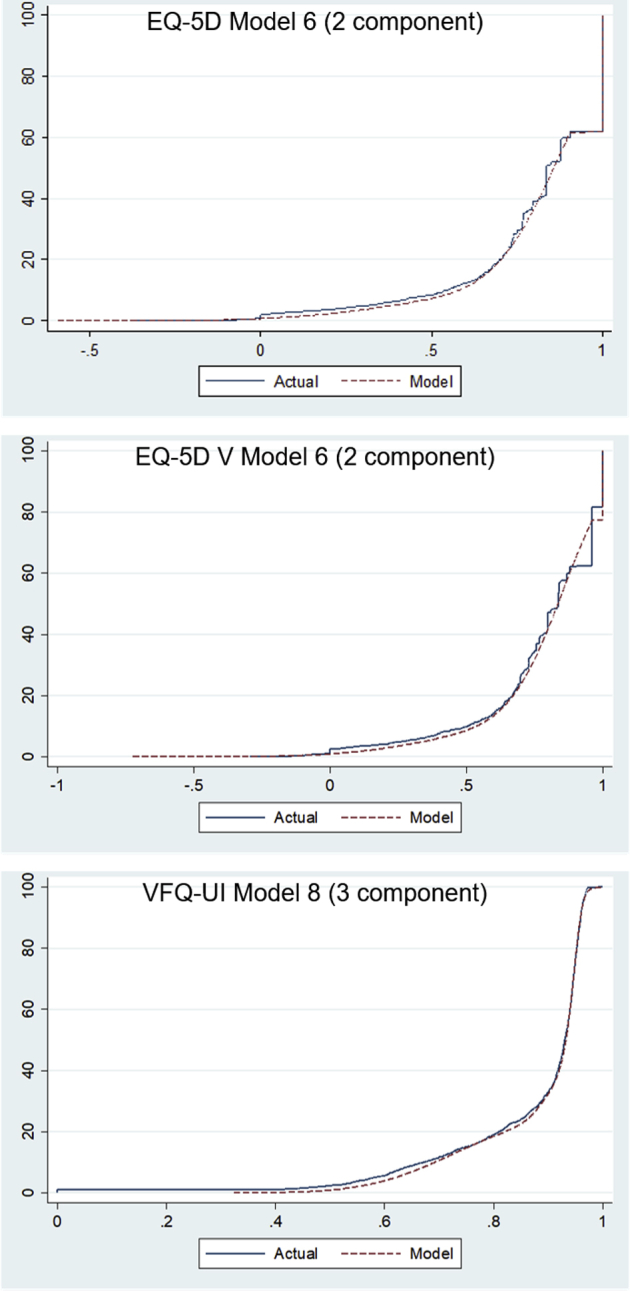
Cumulative distribution function for the simulated data for the 2-component models for the EQ-5D and EQ-5D V and 3-component model for the VFQ-UI.

Figure 3 shows the cumulative distribution functions for the simulated data for the 2-component models for the EQ-5D and EQ-5D V and the 3-component model for the VFQ-UI (all with within-component variables for BSE, WSE, age, and sex and with component membership predicted by BSE and WSE). The cumulative distribution functions for the models with 1 component (not presented here) demonstrated a disparity between the actual and modeled data, which reduced when additional components were added. There was little difference between the actual and modeled data for the 2-, 3-, and 4-component models, so this did not change the decision regarding the best-fitting models.

EQ-5D V indicates EQ-5D with vision bolt-on; VFQ-UI, Visual Functioning Questionnaire-Utility Index.

### Within-Component Means and Probabilities

Table 3 presents the mean utility for each component and the probability that a patient in LEAVO was in that component for each utility measure. The 2-component models for the EQ-5D and EQ-5D V had 1 mean approximately equal to 0.44 and the other approximately equal to 0.85, and increasing the number of components added more means around the higher value. None of the models picked up the probability mass at 0 that is usually observed for EQ-5D. This is likely to be because our models considered component membership as a function of visual acuity alone, but visual acuity is not the only contributing factor to the EQ-5D, as many patients in LEAVO also had other comorbidities, and the EQ-5D and EQ-5D V decreased as the number of comorbidities increased. In the economic model for which the mappings were designed, only visual acuity was modeled and not comorbidities, so the utilities relied on an average number of comorbidities. Using the VFQ-UI, additional components were clustered around 0.8 to 0.9, where the bulk of observations lay.

**Table 3:** Mean utility for each component, and the probability that a patient in LEAVO was in that component for each utility measure.

Utility measure	Number of components	Component	Mean	Probability
EQ-5D	2	1	0.869	.872
		2	0.443	.127
	3	1	0.883	.369
		2	0.839	.535
		3	0.386	.096
	4	1	0.836	.571
		2	0.842	.110
		3	0.498	.092
		4	0.932	.226
EQ-5D-V	2	1	0.442	.142
		2	0.843	.858
	3	1	0.842	.892
		2	0.554	.003
		3	0.428	.134
	4	1	0.742	.446
		2	0.864	.447
		3	0.775	.082
		4	0.330	.081
VFQ-UI	2	1	0.945	.609
		2	0.791	.391
	3	1	0.946	.588
		2	0.735	.250
		3	0.896	.161
	4	1	0.897	.153
		2	0.587	.049
		3	0.946	.590
		4	0.748	.208

EQ-5D V indicates EQ-5D with vision bolt-on; VFQ-UI, Visual Functioning Questionnaire-Utility Index.

## Discussion

We found that the EQ-5D and EQ-5D V were best predicted using 2-component models (the VFQ-UI using 3-component models) where utility within each component was a function of age, sex, and visual acuity in both eyes and the probability of component membership was a function of visual acuity in both eyes. Our mappings were designed for use in an economic model in macular edema secondary to CRVO, to allow utility to be predicted long-term from visual acuity.

We provide mappings for 3 measures of utility. An Excel tool that calculates a patient’s utility using the 3 measures when a user inputs their characteristics is provided at https://figshare.shef.ac.uk/articles/Utility_calculator_for_visual_acuity/9873731/1. As a generic measure of health, the EQ-5D allows comparisons across disease areas and is NICE’s preferred measure of utility for adults.^3^ Nevertheless, previous research has expressed concern regarding the validity of the EQ-5D in visual disorders^22^ and has identified vision as an area in which bolt-ons may be required.^23^ The VFQ-UI has been found to be more sensitive to changes in visual acuity.^14^ Although our analysis does not permit direct comparison of goodness of fit between the different measures, visual inspection confirms that the VFQ-UI appears to more closely align with BSE and WSE BCVA than with the EQ-5D. By developing 3 mappings, we provide options for analysts to use these different measures to estimate utility.

Our analyses found that models with multiple components fitted the data better than those with single components. This is consistent with findings from other disease areas, in which ALDVMMs with multiple components outperform linear regressions using ordinary least squares estimation.^21^^,^^24^ Nevertheless, previous mappings in visual acuity have been limited to ordinary least squares,^14^ and so our analyses provide a valuable addition to the literature.

The inclusion of age and sex within components was both found to improve model fit. These variables are included in economic models as standard, and including them in utility estimation is important for accurately modeling utility over a long time horizon. Consistent with Brazier et al,^14^ but in contrast with Claxton et al,^13^ we found that the inclusion of an interaction term between the BSE and WSE did not improve the model fit.

Although visual acuity was a significant predictor within components and improved model fit for predicting component membership, it did not pick up a separate component for patients with utility at or below 0 for EQ-5D and EQ-5D V. This may be because of the presence of comorbidities within the population, which contributed to lower utility scores. Changing a person’s visual acuity alone would not change their underlying comorbidities and so would not change their probability of belonging to an additional component with different comorbidities. The LEAVO economic model did not specifically include comorbidities and instead implicitly assumed that patients have an average comorbidity profile, reflecting the likely data available to users of the mapping model and in line with good practice guidance.^21^

In patients with CRVO, hypertension is a common comorbidity.^25^^,^^26^ Our mappings are therefore appropriate for use in economic analysis, but we note that the exclusion of comorbidities does limit the accuracy of modeling utility. Macedo et al^27^ found that the EQ-5D-3L utility index is associated with the number of reported comorbidities. Brazier et al^14^ found that including comorbidities improved model fit in predicting both the EQ-5D and VFQ-UI. We were unable to develop a mapping that included comorbidities, as these were recorded only at baseline in the LEAVO study and may have changed over the 100-week trial duration.

A limitation of the robust estimator of the variance used in the statistical model was the inclusion of repeated observations of the same patients to increase the number of observations available. A cluster-robust estimator of the standard errors could have been used, which is robust in the presence of the correlation between observations for each individual. This does not change the estimated coefficients from the ALDVMM and affects only the standard errors used in the probabilistic sensitivity analyses.

It is unclear whether our analyses could be applied in visual disorders other than macular edema secondary to CRVO. We note that some previous economic evaluations have assumed utilities are common across visual disorders, for example, the use of utilities from age-related macular degeneration^28^ in scenario analysis for an economic evaluation in macular edema secondary to CRVO.^16^ Although it may be plausible to assume that the impact on health-related quality of life of changing visual acuity is not dependent on the underlying disease, the absolute utility values may vary across conditions, particularly where comorbidities vary, which would affect quality-adjusted life-year gains from mortality benefits.

## Conclusion

Our mappings can be used to predict the EQ-5D, EQ-5D V, and VFQ-UI from BCVA. Our analyses found that including multiple components in ALDVMMs improved model fit. Our analyses can be used in economic evaluations to predict utility as a function of variables routinely included in economic models for visual disorders, but we note that other comorbidities may also contribute to absolute utility scores.
