# Supplementary material for: Mapping From Visual Acuity to EQ-5D, EQ-5D With Vision Bolt-On, and VFQ-UI in Patients With Macular Edema in the LEAVO Trial
Source: Value Health. 2020 Jul;23(7):928–35. doi: 10.1016/j.jval.2020.03.008 (PMC7427317; doi:10.1016/j.jval.2020.03.008)
Supplement: Appendix Tables 1-3 [file mmc1.docx]

**Appendix**

Supplementary table 1: Spearman correlation coefficients

|  | **EQ-5D** | **EQ-5D V** | **VFQ-UI** |
| --- | --- | --- | --- |
| *Better seeing eye visual acuity* | | | |
| N (pairs) | 2470 | 2321 | 2481 |
| Correlation coefficient* | 0.3055 | 0.2870 | 0.0707 (p=0.0004) |
| *Worse seeing eye visual acuity* | | | |
| N (pairs) | 2472 | 2323 | 2483 |
| Correlation coefficient* | 0.2401 | 0.2546 | 0.1913 |
| *Age* | | | |
| N (pairs) | 2472 | 2323 | 2483 |
| Correlation coefficient* | -0.2684 | -0.2192 | 0.2578 |
| *Sex* | | | |
| N (pairs) | 2472 | 2323 | 2483 |
| Correlation coefficient* | -0.1461 | -0.1191 | -0.0462 (p=0.0213) |

* p<0.0001 unless otherwise stated

EQ-5D V, EQ-5D with vision bolt-on; VFQ-UI, Visual Functioning Questionnaire-Utility Index.

Supplementary table 1: model fit statistics for EQ-5D V

| **Model** | **Number of components** | **Within-component variables** | **Component membership variables** | **Log likelihood** | **AIC** | **BIC** | **Mean error** | **MAE** | **RMSE** |
| --- | --- | --- | --- | --- | --- | --- | --- | --- | --- |
| 1 | 1 | BSE, WSE, age, sex | NA | -317.7337 | 647.4674 | 681.9659 | 0.0100325 | 0.1536135 | 0.20771018 |
| 2 | 1 | BSE, WSE, BSE*WSE, age, sex | NA | -317.719 | 649.4381 | 689.6863 | 0.0100193 | 0.1536028 | 0.20770385 |
| 3 | 2 | BSE, WSE, age, sex | Constant | -78.42069 | 182.8414 | 257.5882 | 0.0003302 | 0.1522023 | 0.20811671 |
| 4 | 2 | BSE, WSE, BSE*WSE, age, sex | Constant | -77.06003 | 184.1201 | 270.3664 | 0.000372 | 0.1520822 | 0.20796268 |
| 5 | 2 | BSE, WSE, age, sex | BSE | -55.7529 | 139.5058 | 220.0023 | 0.0014217 | 0.1516238 | 0.20681272 |
| 6 | 2 | BSE, WSE, age, sex | BSE, WSE | -51.0631 | 132.1262 | 218.3725 | 0.0014786 | 0.1514141 | 0.20651357 |
| 7 | 3 | BSE, WSE, age, sex | Constant | -55.41668 | 150.8334 | 265.8284 | 0.0008968 | 0.1521651 | 0.20806247 |
| 8 | 3 | BSE, WSE, age, sex | BSE, WSE | -23.25709 | 94.51419 | 232.5083 | 0.0012605 | 0.1514113 | 0.20656355 |
| 9 | 4 | BSE, WSE, age, sex | Constant | -40.23112 | 134.4622 | 289.7056 | 0.0013342 | 0.1522218 | 0.20803716 |
| 10 | 4 | BSE, WSE, age, sex | BSE, WSE | -11.17183 | 88.34366 | 278.0855 | 0.0013407 | 0.1487271 | 0.20432547 |

AIC, Akaike information criteria; BIC, Bayesian information criteria; BSE, better seeing eye visual acuity; EQ-5D V, EQ-5D with vision bolt-on; MAE, mean absolute error; NA, not applicable; RMSE, root mean square error; WSE, worse seeing eye visual acuity.

Supplementary table 2: model fit statistics for VFQ-UI

| **Model** | **Number of components** | **Within-component variables** | **Component membership variables** | **Log likelihood** | **AIC** | **BIC** | **Mean error** | **MAE** | **RMSE** |
| --- | --- | --- | --- | --- | --- | --- | --- | --- | --- |
| 1 | 1 | BSE, WSE, age, sex | NA | 2115.292 | -4218.584 | -4183.685 | 0.0082523 | 0.0784709 | 0.10328809 |
| 2 | 1 | BSE, WSE, BSE*WSE, age, sex | NA | 2129.159 | -4244.318 | -4203.604 | 0.0078868 | 0.0781374 | 0.10288856 |
| 3 | 2 | BSE, WSE, age, sex | Constant | 2115.292 | -8574.639 | -8499.025 | 0.008252 | 0.0784707 | 0.10328806 |
| 4 | 2 | BSE, WSE, BSE*WSE, age, sex | Constant | 4301.412 | -8572.824 | -8485.578 | -0.0050565 | 0.0792015 | 0.1083911 |
| 5 | 2 | BSE, WSE, age, sex | BSE | 4398.724 | -8769.448 | -8688.018 | -0.0012497 | 0.0745142 | 0.10292355 |
| 6 | 2 | BSE, WSE, age, sex | BSE, WSE | 4432.405 | -8834.81 | -8747.564 | 0.0006735 | 0.0741814 | 0.10220785 |
| 7 | 3 | BSE, WSE, age, sex | Constant | 4464.795 | -8889.589 | -8773.261 | -0.005855 | 0.0793793 | 0.10929272 |
| 8 | 3 | BSE, WSE, age, sex | BSE, WSE | 4605.025 | -9162.051 | -9022.457 | -0.0005292 | 0.07372 | 0.10221405 |
| 9 | 4 | BSE, WSE, age, sex | Constant | 4522.183 | -8990.367 | -8833.323 | -0.0057675 | 0.0790525 | 0.10884583 |
| 10 | 4 | BSE, WSE, age, sex | BSE, WSE | 4632.919 | -9199.837 | -9007.895 | -0.0002894 | 0.0735843 | 0.10204516 |

AIC, Akaike information criteria; BIC, Bayesian information criteria; BSE, better seeing eye visual acuity; MAE, mean absolute error; NA, not applicable; RMSE, root mean square error; VFQ-UI, visual functioning questionnaire-utility index; WSE, worse seeing eye visual acuity.
